# Device-detektiertes Vorhofflimmern – was nun? Executive Summary des DGK-Positionspapiers

**DOI:** 10.1007/s00399-025-01112-7

**Published:** 2025-10-29

**Authors:** Tobias Tönnis, Nina Becher, Ralf Birkemeyer, David Duncker, Lars Eckardt, Klaus Gröschel, Dominik Linz, Christian Meyer, Florian Straube, Arian Sultan, Rolf Wachter, Maura Zylla, Paulus Kirchhof

**Affiliations:** 1 Klinik für Kardiologie, Universitätsklinikum Hamburg-Eppendorf, Universitäres Herz- und Gefäßzentrum, Martinistraße 52, 20246 Hamburg, Deutschland; 2 Herzklinik Ulm, Ulm, Deutschland; 3 Hannover Herzrhythmus Centrum, Klinik für Kardiologie und Angiologie, Medizinische Hochschule Hannover, Hannover, Deutschland; 4 Klinik für Kardiologie II, Universitätsklinikum Münster (UKM), Münster, Deutschland; 5 Klinik und Poliklinik für Neurologie, Universitätsmedizin der Johannes Gutenberg-Universität Mainz, Mainz, Deutschland; 6 Maastricht UMC Heart + Vascular Center Department of Cardiology, Maastricht, Niederlande; 7 Evangelisches Krankenhaus Düsseldorf Klinik für Kardiologie, Düsseldorf, Deutschland; 8 Klinik für Kardiologie und Internistische Intensivmedizin, München Klinik Bogenhausen, München, Deutschland; 9 Abteilung für Kardiologie, Asklepios Klinik St. Georg, Hamburg, Deutschland; 10 Universitätsklinikum Leipzig Klinik und Poliklinik für Kardiologie, Leipzig, Deutschland; 11 Kardiologie, Angiologie u. Pneumologie, Universitätsklinikum Heidelberg Klinik für Innere Med. III, Heidelberg, Deutschland; 12 Kommission für Klinische Kardiovaskuläre Medizin, Deutsche Gesellschaft für Kardiologie, Düsseldorf, Deutschland

**Keywords:** Vorhofflimmern, Antikoagulation, Herz und Hirn, Herzschrittmacher, ICD, Atrial fibrillation, Anticoagulation, Heart and brain, Pacemaker, ICD

## Abstract

Device-detektiertes Vorhofflimmern (DDAF) ist wie klinisches Vorhofflimmern (AF) ebenfalls mit einem erhöhten Risiko für thrombembolische Ereignisse assoziiert, wobei das Risiko jedoch deutlich geringer auszufallen scheint. Die Wirksamkeit und Sicherheit einer oralen Antikoagulation bei DDAF wurden in zwei großen randomisierten Studien (NOAH-AFNET 6 und ARTESIA [13, 19]) untersucht. Es zeigte sich eine niedrige Rate an ischämischen Schlaganfällen ohne Antikoagulation (etwa 1 % pro Jahr). Durch eine therapeutische Antikoagulation kann dieses Risiko noch gesenkt werden, allerdings steigt dabei die Gefahr schwerer Blutungen. In dem kürzlich veröffentlichten Positionspapier [36] der Deutschen Gesellschaft für Kardiologie wurde die aktuelle Studienlage dargestellt und Expertenempfehlungen zum Umgang mit DDAF gegeben.

## Abstract (en)

Device-detected atrial fibrillation (DDAF), like clinical atrial fibrillation (AF), is also associated with an increased risk of thromboembolic events, although the risk appears to be significantly lower. The efficacy and safety of oral anticoagulation in DDAF were investigated in two large randomized trials (NOAH-AFNET 6 and ARTESIA [13, 19]). They showed a low rate of ischemic strokes without anticoagulation (about 1% per year). This risk can still be reduced by therapeutic anticoagulation, but the risk of severe bleeding increases. In the recently published position paper [36] of the German Society of Cardiology, the current study situation was presented and expert recommendations were given on how to deal with DDAF.

Bei Device-detektiertem Vorhofflimmern (DDAF) handelt es sich um kurze, meist asymptomatische Episoden von Vorhofflimmern (AF), die in implantierten Devices wie Herzschrittmachern, Defibrillatoren, CRT-Geräten (kardiale Resynchronisationstherapie) oder Ereignisrekordern registriert werden können (Abb. 1). Dies ist zu unterscheiden von Vorhofflimmern, welches in einem Oberflächen-EKG dokumentiert wurde. Device-detektiertes Vorhofflimmern (DDAF) wurde bisher auch als subklinisches Vorhofflimmern (SCAF) oder atriale Hochfrequenzepisoden (AHRE) bezeichnet. Bisher umfassen diese Definitionen keine Vorhofarrhythmien, die mittels Wearables wie Smartwatches aufgezeichnet wurden.

**Abb. 1:**
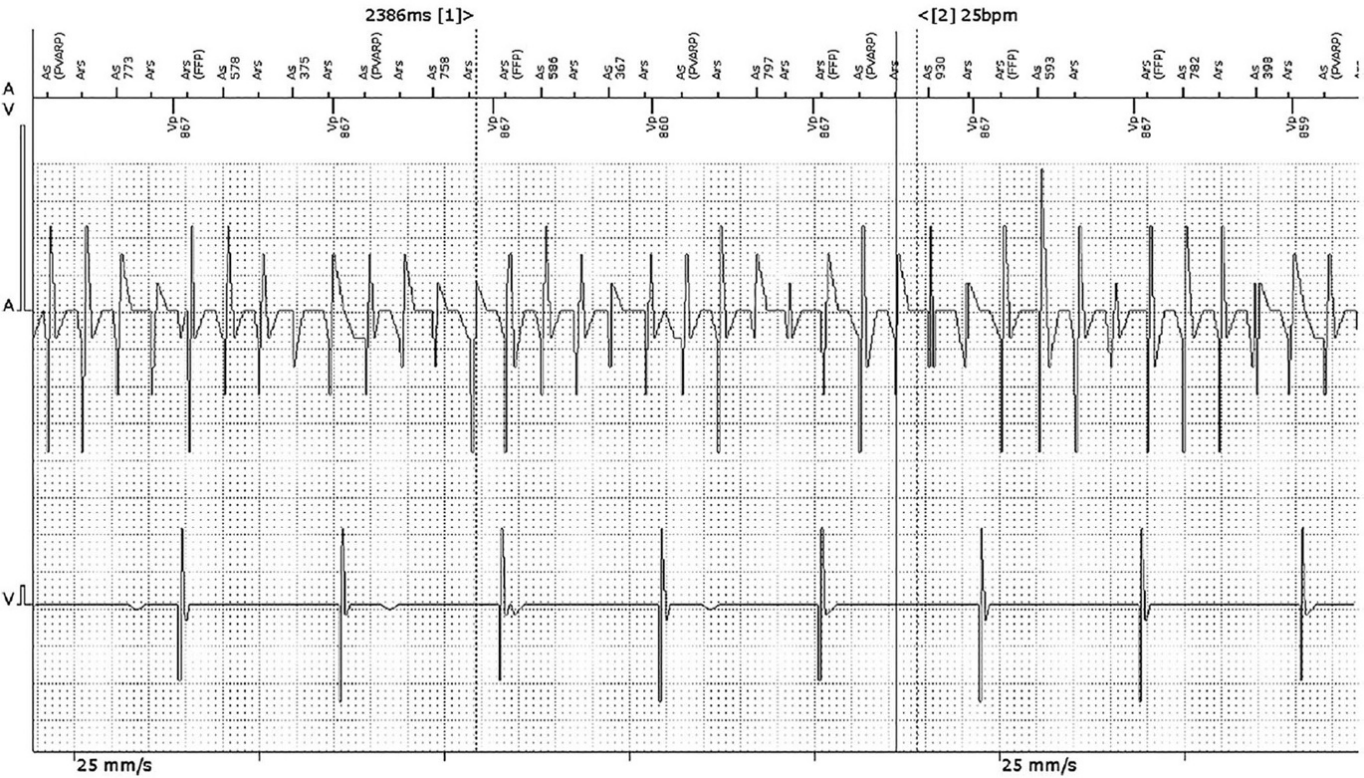
Gespeichertes Elektrogramm (EGM) eines DDD-Schrittmachers mit Device detektiertem Vorhofflimmern. (Aus [36])

## DDAF und Thrombembolierisiko

DDAF bei Patienten ohne bekanntes AF ist mit einem leicht erhöhten Thrombembolierisiko verbunden, dieses ist deutlich niedriger ist als bei im Oberflächen-EKG dokumentierten AF [23]. Die ASSERT-Studie zeigte, dass Patienten, bei denen DDAF innerhalb der ersten 3 Monate auftrat, ein 2,5-fach höheres Schlaganfallrisiko hatten als Patienten ohne DDAF [12]. Das Risiko stieg vor allem bei Episoden, die länger als 24 h dauerten [39]. Allerdings wurde in ASSERT nur sporadisch nach EKG-dokumentiertem AF gesucht.

Die LOOP-Studie hat die Prävalenz und klinische Bedeutung von DDAF bei älteren Menschen untersucht [33]. Dabei erhielten 25 % der 6004 Probanden im Alter zwischen 70 und 90 Jahren einen implantierbaren Ereignisrekorder. Innerhalb von 5 Jahren wurde bei 32 % der mit Rekorder überwachten Teilnehmer DDAF festgestellt, in der anderen Gruppe nur bei 12 %. Trotz höherer Antikoagulationsraten bei Patienten mit Ereignisrekorder (30 % vs. 13 %) waren die Schlaganfallraten mit etwa 4,5 % bzw. 5,6 % nicht signifikant unterschiedlich. Diese Ergebnisse sind im Einklang mit der begrenzten Effektivität von AF-Screening-Studien wie STROKESTOP [35] und STROKESTOP II [17].

## Bedeutung der Vorhofflimmerlast

Die AF-Last (AF-Burden) beschreibt den Anteil der Zeit, in welcher bei Patienten Vorhofflimmern besteht, im Verhältnis zur gesamten Monitorperiode [21]. Bei Patienten mit klinischem, paroxysmalem AF und implantierten Devices liegt die durchschnittliche AF-Last bei etwa 10 % [4]. Die AF-Last kann durch rhythmuserhaltende Therapie gesenkt werden. Die Detektion von DDAF hängt stark vom Beobachtungszeitraum ab. Viele Patienten haben jedoch nur wenige kurze Episoden; in der LOOP-Studie lag die mediane DDAF-Last bei 0,13 % [33].

**Abb. 2:**
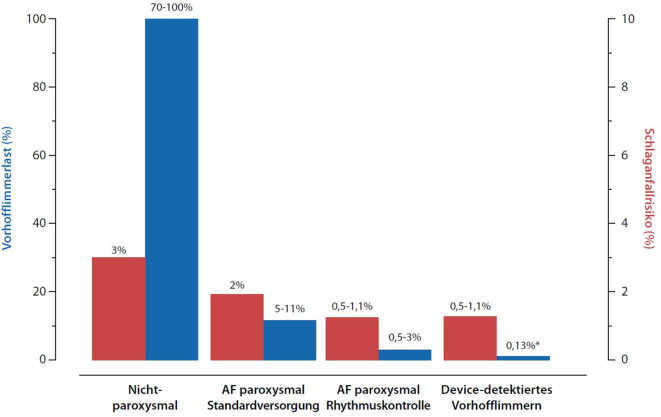
Konzeptioneller Zusammenhang zwischen AF-Last (AF-Burden) und Schlaganfallsrisiko. Die vorhandenen Daten legen nahe, dass eine invers-exponentielle Beziehung besteht. Mutmaßlich sinkt das Schlaganfallsrisiko ohne Antikoagulation um 1 %/Jahr (oder 20–30 %), wenn sich die Vorhofflimmerlast 10-fach reduziert. Bei sehr niedriger Vorhofflimmerlast ist bei exponentiellem Zusammenhang zu erwarten, dass keine klinisch erfassbare weitere Abnahme des Schlaganfallsrisikos vorliegt. (Aus [2])

Ein Zusammenhang zwischen AF-Last und Schlaganfallrisiko scheint wahrscheinlich. Ischämische Schlaganfälle treten bei paroxysmalem AF seltener auf als bei persistierendem oder permanentem Vorhofflimmern([2, 5]; Abb. 2). So beträgt das ungefähre jährliche Risiko für ischämische Schlaganfälle bei nichtparoxysmalem Vorhofflimmern 3 %. Frühe rhythmuserhaltende Therapien reduzieren das Schlaganfallrisiko bei Risikopatienten (CHA2DS2-VASc ≥ 2) weiter [18], wobei das Vorliegen von Sinusrhythmus entscheidend zu sein scheint [8].

## Therapeutische Konsequenzen bei DDAF

### Antikoagulation bei Patienten mit DDAF ohne EKG-Dokumentation von AF?

Sehr kurze Episoden von DDAF können nur schwer von Artefakten unterschieden werden. Daher liegen randomisierte Studiendaten nur für DDAF-Episoden ≥ 6 min Dauer vor. Eine erste randomisierte Studie (IMPACT) zeigte 2015 keine relevante Reduktion von Schlaganfällen durch eine Antikoagulation bei DDAF [24]. Kürzlich wurden zwei große randomisierte Studien veröffentlicht, die den Einsatz von DOAC bei Patienten mit DDAF ≥ 6 min ohne vorher bekanntes AF untersuchten:

In der NOAH-AFNET 6-Studie [19] wurden über 2500 Patienten ab 65 Jahren mit DDAF und Risikofaktoren zu Edoxaban oder Placebo (bzw. ASS bei bestehender Indikation) randomisiert. Nach einer medianen Nachbeobachtungszeit von 21 Monaten wurde die Studie wegen Sicherheitsbedenken vorzeitig beendet, da schwere Blutungen (2,1 % gegenüber 1,0 %; HR 2,10 [95 % KI 1,30–3,38]; *p* = 0,002) doppelt so häufig auftraten, während der primäre Effektivitätsendpunkt (kardiovaskulärer Tod, Schlaganfall, systemische Embolie) keinen signifikanten Unterschied zeigte. Die Schlaganfallrate lag in der Placebo-Gruppe bei 1,1 % pro Jahr, in der Edoxaban-Gruppe bei 0,9 %. Insgesamt war die Anzahl der Schlaganfälle aber trotz Komorbiditäten (Median CHA2DS2-VASc 4) gering (22 vs. 27).

Die ARTESIA-Studie [13] mit 4012 randomisierten Patienten mit DDAF von 6 min bis 24 h Dauer zeigte, dass Apixaban nach 3,5 Jahren im Vergleich zu Aspirin das Risiko für Schlaganfälle und Embolien signifikant senkte (0,8 % vs. 1,2 %, HR 0,63; *p* = 0,007). Allerdings traten unter Apixaban wie erwartet mehr schwere Blutungen auf (1,7 % gegenüber 0,9 %; HR 1,80 [95 % KI, 1,26–2,57]; *p* < 0,001).

Auch wenn die Ergebnisse von NOAH-AFNET 6 und ARTESIA unterschiedlich scheinen, sind sie doch konsistent [25]. Insbesondere besteht bei beiden Studien eine niedrige Rate ischämischer Schlaganfälle von etwa 1 % pro Jahr bei Patienten ohne Antikoagulation.

Eine Antikoagulation bei Patienten mit DDAF reduzierte das Auftreten von zerebralen Ischämien um 32 %, erhöhte aber gleichzeitig die Rate schwerer Blutungen um 62 %. Aufgrund der niedrigen Schlaganfallrate ist der absolute Nutzen gering (0,3 % pro Jahr Therapie). In ARTESIA ergibt sich eine „number needed to treat“ (NNT) von 217, um einen Schlaganfall pro Jahr zu verhindern, und über 300, um einen schweren Schlaganfall (Rankin Score 3–6) zu vermeiden [43].

### Potenzielle Risikogruppen

Subgruppenanalysen aus NOAH AFNET 6 und ARTESIA können dabei unterstützen, die Patienten mit DDAF zu identifizieren, bei denen eine Antikoagulation am ehesten sinnvoll sein könnte. Allerdings können solche Analysen nur hypothesengenerierend sein und bedürfen der weiteren Prüfung.

#### Lange Episodendauer

Das Thrombembolierisiko bei DDAF kann je nach Episodendauer und CHA_2_DS_2_-VASc-Score variieren [16, 39]. In ARTESIA zeigte sich, dass die Dauer der längsten DDAF-Episode keinen Einfluss auf das Schlaganfallrisiko oder die Wirksamkeit der Antikoagulation hatte [26], allerdings wurden keine Patienten mit Episoden über 24 h eingeschlossen. In NOAH-AFNET 6 hatten 11 % der Patienten Episoden von ≥ 24 h Dauer [3]. Bei dieser Gruppe war die Schlaganfallrate mit 1 % pro Jahr ähnlich wie bei Patienten mit kürzeren Episoden und die Antikoagulation zeigte keinen signifikanten Effekt. Ein Grund für die niedrigen Ereignisraten auch in dieser Subgruppe ist vermutlich, dass Patienten mit im EKG detektiertem AF im Studienverlauf (29 % bei Patienten mit Episoden ≥ 24 h) leitliniengerecht antikoaguliert und von der Analyse ausgeschlossen wurden.

#### CHA_2_DS_2_-VASc-Score 5–9

Die aktuellen Empfehlungen in den Leitlinien sehen vor, bei Patienten mit DDAF eine orale Antikoagulation in Betracht zu ziehen, sofern relevante Risikofaktoren für einen Schlaganfall bestehen [40]. Die Entscheidung basiert auf Schlaganfallrisiko-Scores, die für EKG-dokumentiertes AF entwickelt wurden [14, 39, 40]. Ob dies auch bei DDAF anwendbar ist, ist nicht belegt.

NOAH-AFNET 6 und ARTESIA untersuchten in Subanalysen die Wirksamkeit und Sicherheit der Antikoagulation bei unterschiedlichem CHA_2_DS_2_-VASc-Score [22]. Die Subanalyse von NOAH-AFNET 6 deutet nicht darauf hin, dass Edoxaban bei einem Score > 4 wirksamer ist als bei einem CHA_2_DS_2_-VASc-Score < 4. Es besteht eher ein Hinweis auf erhöhtes Blutungsrisiko bei multiplen Komorbiditäten. Die ARTESIA-Analyse zeigt allerdings bei Scores > 4 einen Vorteil für Apixaban gegenüber ASS, mit weniger thrombembolischen Ereignissen bei vergleichbarem Blutungsrisiko [32]. Allerdings wurden in ARTESIA alle Patienten in der Kontrollgruppe mit Aspirin behandelt.

Zusammengefasst: Für DDAF-Patienten mit CHA_2_DS_2_-VASc-Score > 4 kann eine Antikoagulation derzeit nicht generell empfohlen werden. Umgekehrt ist es aber sehr unwahrscheinlich, dass eine Antikoagulation bei DDAF und CHA_2_DS_2_-VASc-Score von 2–4 sinnvoll ist.

Eine grundsätzliche Empfehlung bei erhöhtem CHA_2_DS_2_-VASc-Score kann nur bei im EKG nachgewiesenem AF erfolgen. Es ist daher unbedingt sinnvoll, bei Patienten mit DDAF regelmäßig (z. B. alle 6 Monate) ein Oberflächen-EKG durchzuführen. Mit diesem Vorgehen konnte in NOAH-AFNET 6 und ARTESIA bei ca 10 % der Studienteilnehmer AF dokumentiert werden, bei welchen dann eine Antikoagulation eingeleitet wurde.

#### Patienten nach Schlaganfall/TIA

Patienten nach einem Schlaganfall haben ein erhöhtes Risiko für erneute ischämische Ereignisse [31]. Eine Antikoagulation kann dieses Risiko um 60–70 % senken, aber nicht alle Schlaganfälle sind dadurch vermeidbar [15]. In der MOnDAFIS-Studie führte die stationäre Rhythmusüberwachung (durchschnittlich fünf Tage) zwar zu einer häufiger initiierten Antikoagulation, konnte aber erneute Schlaganfälle nicht verhindern [11, 37]. Längere Detektionszeiten erhöhen die Wahrscheinlichkeit, Vorhofflimmern zu erkennen und die Antikoagulation zu steigern. Die Find-AF2-Studie untersucht derzeit, ob intensiveres Monitoring erneute Schlaganfälle verhindern kann [38].

In der NOAH-AFNET-6-Studie zeigte eine präspezifizierte Analyse von Patienten mit DDAF und Schlaganfall oder TIA in der Vorgeschichte keinen Effekt der Antikoagulation auf das Auftreten von Schlaganfall, Embolie oder kardiovaskulärem Tod (primärer Endpunkt; [7]). Die Schwere der Schlaganfälle war mit oder ohne Antikoagulation ähnlich. Eine Antikoagulation führte erwartungsgemäß aber zu mehr schweren Blutungsereignissen.

In der ARTESIA-Studie hatten 8,6 % der Patienten (346 Personen) Schlaganfälle oder TIA in der Vorgeschichte [32]. Das absolute Risiko für Schlaganfall oder systemische Embolie wurde durch Apixaban über einen Zeitraum von 3,5 Jahren um 7 % reduziert (versus 1 % bei Patienten ohne Vorgeschichte von Schlaganfällen oder TIA), das relative Risiko um 60 %. Fatale oder stark einschränkende Schlaganfälle wurden um drei Viertel reduziert. Aber es verdoppelte sich das Risiko für schwere Blutungen gegenüber Patienten unter Aspirin. 90 % der Blutungen konnten allerdings konservativ behandelt werden.

Zusammenfassend sind Patienten mit DDAF und einem Schlaganfall/TIA in der Vorgeschichte eine Patientengruppe, bei der eine Antikoagulation in Betracht gezogen werden kann.

#### Rolle von „vaskulären Erkrankungen“ bei DDAF

Eine kombinierte präspezifizierte Subgruppenanalyse von Patienten mit und ohne manifeste Gefäßerkrankung der NOAH-AFNET 6- und ARTESIA-Studien zeigt, dass bei Patienten mit DDAF ohne vaskuläre Erkrankung nur wenige Schlaganfälle auftreten und eine Antikoagulation keinen klaren Nutzen hat, aber mehr Blutungen verursacht [30]. Eine Gefäßerkrankung wurde definiert als ein stattgehabter Schlaganfall und/oder eine bekannte koronare oder periphere Gefäßerkrankung.

Bei Patienten mit vaskulärer Erkrankung traten häufiger Schlaganfälle auf, und eine Antikoagulation reduzierte die Schlaganfallsrate (1,3 % vs. 0,7 % in NOAH AFNET 6; 1,5 % vs. 0,7 % in ARTESIA). Auch bei den primären Endpunkten beider Studien war die Antikoagulation bei Patienten mit Gefäßerkrankung tendenziell effektiver (Interaktions-*p*-Wert = 0,08).

Diese Ergebnisse legen nahe, dass bei Patienten mit Nachweis von DDAF ohne begleitende Gefäßerkrankung eine Antikoagulation nicht durchgeführt werden sollte. Wenn eine vaskuläre Erkrankung mit Indikation für Aspirin vorliegt, kann jedoch ein Wechsel von Aspirin auf ein DOAC eher erwogen werden.

### Interventionelle Thrombembolieprophylaxe bei DDAF

Die Entscheidung für eine orale Antikoagulation bei DDAF ist mitunter komplex. Alternativen wie der Vorhofohrverschluss (LAAO) könnten das Risiko thromboembolischer Ereignisse senken und Blutungsrisiken reduzieren. Daten für Patienten mit DDAF liegen dazu nicht vor. Die chirurgische Entfernung des linken Vorhofohrs während Herzoperationen verringert das Schlaganfallsrisiko bei AF-Patienten [44]. Die OPTION-Studie zeigt eine niedrige Schlaganfallsrate (ca. 0,4 %/Jahr) nach interventionellem Vorhofverschluss bei Patienten mit geringer AF-Last nach Ablation [42]. Aber ob die potenziellen prozeduralen und postprozeduralen Risiken der LAAO diese Vorteile rechtfertigen, ist jedoch noch unklar, da kontrollierte Studien fehlen.

### Rhythmuserhaltende Therapie (antiarrhythmische Therapie, Ablation)

Eine frühzeitige Rhythmuskontrolle bei Patienten mit klinischem AF und kardiovaskulären Risikofaktoren kann kardiovaskuläre Ereignisse verhindern [18] und das Fortschreiten des atrialen Remodelings sowie die Chronifizierung von AF reduzieren [9, 20]. Die EAST-AFNET 4-Studie zeigte bei solchen Patienten einen Vorteil einer frühen Rhythmuskontrolle, unabhängig von Symptomen [45].

Da bei DDAF per Definition Sinusrhythmus im EKG vorliegt und die AF-Last meist niedrig ist, ist kritisch zu hinterfragen, ob eine rhythmuserhaltende Therapie auch bei DDAF prognostische Vorteile bietet, insbesondere wegen möglicher Nebenwirkungen antiarrhythmischer Medikamente und Komplikationsmöglichkeiten durch die Ablation. Aber die Detektion von DDAF kann Anlass sein, frühzeitig sinnvolle Lebensstilveränderungen anzuregen. Vor einer Therapie sollten Risiko- und Lifestyle-Faktoren optimiert werden, um atriales Remodeling zu verzögern und den Rhythmuserhalt zu fördern (siehe AWMF S3 Leitlinie Vorhofflimmern).

## DDAF nach Katheterablation von Vorhofflimmern

Ereignisrekorder, die nach AF-Ablation zu Studienzwecken implantiert wurden, zeigen eine niedrige AF-Last (< 1 % im Median über 3 Jahre), etwas niedriger als bei antiarrhythmischer Therapie (2,4 %) [1]. Die OPTION-Studie bestätigte eine niedrige Schlaganfallsrate nach Ablation [42]. DDAF nach Katheterablation bei Vorhofflimmern hat zum heutigen Stand keine Konsequenzen auf die Antikoagulation. Ob eine Antikoagulation bei der sehr niedrigen AF-Last nach Ablation fortgeführt werden sollte, wird aktuell u. a. in der OCEAN-Studie untersucht [41].

## DDAF-Detektion durch digitale Devices

Digitale Geräte – sog. „consumer electronics“ oder „wearables“ – ermöglichen es, den Herzrhythmus über längere Zeit zu überwachen und Vorhofflimmern (AF) zu erkennen [34]. Diese Geräte können entweder auf PPG (Photoplethysmographie) oder EKG (Elektrokardiogramm) basieren [10, 27, 28], beide Methoden sind in der Lage, längere AF-Episoden erfolgreich zu erkennen.

AF, das mittels Consumer Electronics detektiert wurde, nimmt eine Mittelstellung zwischen im EKG dokumentiertem AF und DDAF ein. Obwohl viele dieser Geräte eine kontinuierliche Überwachung des Herzrhythmus erlauben, werden sie in der Regel nicht ständig getragen – zum Beispiel beim Sport oder nachts. Andere Geräte, wie Smartphone-Apps oder Handheld-Devices, werden nur intermittierend benutzt, etwa 3‑mal am Tag.

**Abb. 3:**
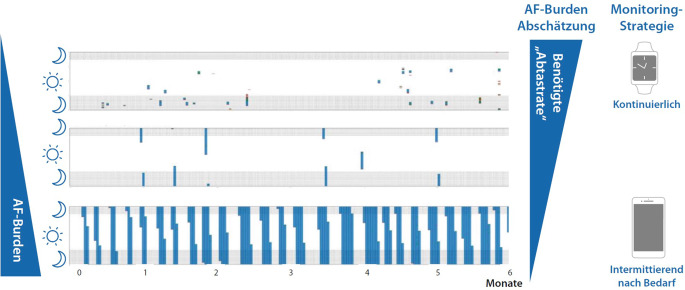
Zusammenhang zwischen erwarteter AF-Last und der Monitoringstrategie. Beispiele für Patienten mit kurzen, mittellangen und langen AF-Episoden (von oben nach unten) während einer 6‑monatigen Nachbeobachtung (X-Achse). Jeder Tag wird durch einen Balken dargestellt, wobei laufende Episoden von AF *blau* dargestellt sind. *Schattierte Bereiche* kennzeichnen die Nacht. *Rechts* Darstellung der benötigten „Abtastrate“ und Monitoring Strategie (kontinuierlich bis intermittierend nach Bedarf) zur Abschätzung der AF-Last (AF-Burden). (Mod. nach [6]; aus [36])

Trotz der unterschiedlichen Überwachungsdauer und -frequenz kann man die AF-Belastung abschätzen ([29]; Abb. 3). Es ist jedoch oft schwierig, die genaue Dauer der AF-Last zu bestimmen, die durch diese Consumer Electronics erkannt wurde.

**Abb. 4:**
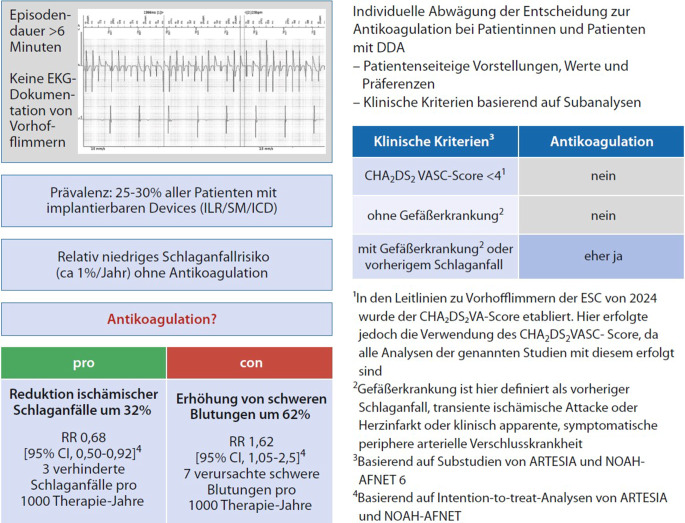
Entscheidungshilfe zur Antikoagulation bei Device-detektiertem Vorhofflimmern (DDAF). (Aus [36])

Das DGK-Positionspapier zur Wearable-basierten Detektion von Arrhythmien beschreibt drei Szenarien für den Einsatz von Wearables bei der Arrhythmiedetektion: 1) als Eventrekorder, 2) aktives Screening und 3) passives Screening. Obwohl Studien wie STROKESTOP [35], LOOP [33] und MonDAFIS [11] zeigen, dass Consumer Electronics zum AF-Screening genutzt werden können, gibt es wenig Evidenz zur Indikationsstellung einer Antikoagulation (siehe AWMF S3-Leitlinie). Die Wirksamkeit von Antikoagulanzien bei mit Wearables erkanntem AF ist bislang nicht in kontrollierten Studien belegt. Wenn AF mit Wearables detektiert wird, sollte es vor allem bei langfristiger Rhythmusüberwachung und niedriger AF-Last am ehesten wie DDAF behandelt werden.

## Fazit für die Praxis

Für Entscheidungshilfen zur Antikoagulation bei Device-detektiertem Vorhofflimmern (DDAF) schauen Sie bitte unter Abb. 4.
